# Toxicity of Polystyrene Microplastics with Cadmium on the Digestive System of *Rana zhenhaiensis* Tadpoles

**DOI:** 10.3390/toxics12120854

**Published:** 2024-11-26

**Authors:** Ye Tang, Xueyi Wu, Yuting Pang, Shimin Xiao, Lei Xie, Yongpu Zhang

**Affiliations:** 1 Life and Environmental Science College, Wenzhou University, Wenzhou 325003, China; 2 Zhejiang Provincial Key Laboratory for Subtropical Water Environment and Marine Biological Resources Protection, Wenzhou University, Wenzhou 325003, China

**Keywords:** microplastics, heavy metal, combined toxicity, *Rana zhenhaiensis* tadpoles, intestinal microbiome, liver

## Abstract

Microplastics pollution in freshwater systems is attracting increasing attention. However, our knowledge of its combined toxicity with heavy metals is scarce. In this study, *Rana zhenhaiensis* was used as the model animal to study the combined poisoning mechanism of cadmium or microplastics on the digestive systems of tadpoles in freshwater. Results showed that the exposure to cadmium and polystyrene increased the mortality and metamorphosis rate of *R. zhenhaiensis* tadpoles, and delayed their growth and development. Cadmium was detected in the livers and intestines, while polystyrene mainly accumulated in the gills and intestines of tadpoles. The individual exposure of cadmium or polystyrene can cause pathological damage to liver tissue, induce oxidative stress in liver, and change gene expression. Cadmium co-exposure with polystyrene can reduce the cadmium accumulation in the liver. While polystyrene can slightly increase cadmium accumulation in the intestine. Exposure to cadmium and polystyrene altered the abundance and community structure of intestinal microbiota, and polystyrene increased the dysregulation of the gut microbiome. In this study, the combined exposure of microplastics and cadmium had a negative impact on *R. zhenhaiensis* tadpoles, but the introduction of microplastics on the toxicity of cadmium on the tadpoles needs further investigation, due to the different characteristics of microplastics.

## 1. Introduction

Plastics are widely used in industrial production due to their good processing properties and stable performance. Over the past few decades, there has been an astonishing surge in plastic production, with global plastic output soaring to 413.8 million tons in 2023 [1]. Driven by the relentless pursuit of high consumer demand and expanded consumption, this growth has been steady, signifying that we now inhabit a world inundated with plastics [2]. Unfortunately, the recycling rate for disposable plastic items is appallingly low, generating an enormous quantity of plastic waste [3]. Being easily damaged and carelessly discarded, this waste plastic finds its way into the environment, precipitating gravely concerning plastic pollution issues [4].

Microplastics (MPs) represent a novel class of emerging contaminants. There are many types of MPs, with polyethylene terephthalate (PET), polyethylene (PE), polystyrene (PS), and polypropylene (PP) being the most commonly detected MPs in aquatic environments [5]. They are widely coming from industry and agriculture, such as microplastic particles contained in cosmetics, plastic and resin particles used as industrial raw materials, or agricultural plastic film [5]. In addition, MPs are generated when plastic waste is broken down under the influence of weathering, mechanical forces, or chemical stress [6], yielding fragments that are further reduced in size by solar radiation and erosion. This intricate process results in the formation of extremely fine particles [7], which are termed MPs when their diameter measures less than 5 mm [8,9]. The National Oceanic and Atmospheric Administration (NOAA) currently defines MPs as minuscule, widespread plastic particles with a diameter of less than 5 mm [10]. These MPs primarily stem from industrial and agricultural activities [11,12]. Due to their stability, recalcitrance, small size, and propensity for displacement, MPs are transported globally via runoff, wind, and ocean currents, pervading all corners of the Earth’s environment [13,14,15,16]. Pollution from MPs has been detected in both marine and terrestrial ecosystems, from the equator to the poles, and in various biotic and abiotic matrices [17], including human beings [18].

Studies suggest that the average concentration of MPs in global rivers is approximately 40 to 50 times greater than the highest observed ocean concentrations [19]. Land-based sources contribute significantly to microplastic loads in the marine environment, with fluxes ranging from 64% to 90% of the total plastic debris. Annually, global rivers transport plastic debris (MPs and large plastics) to oceans at a total amount of 0.41 × 10^6^~4 × 10^6^ t [20]. The abundance of MPs in the southeast bay of Zhejiang Province in China reached 8.9 per m^3^ [21]. In lake water, the abundance is even higher, with 1064 MPs per m^3^ in Poyang Lake and 3400 MPs per m^3^ in Taihu Lake [22]. MPs have also been detected in multiple other countries [23,24], indicating extensive distribution and global concern [25,26]. Their impact on aquatic organisms is significant, causing adverse effects on growth, development, reproduction, and cell functions.

While invisible to the naked eye, the impact of MPs on aquatic organisms is far from negligible. The MPs in water will be ingested by aquatic organisms through swallowing and breathing, accumulate in the body, which causes adverse effects on growth, development, reproduction and other aspects of organisms [27]. For example, zebrafish (*Danio rerio*) presented abnormal phenomena such as mucosal damage, inflammation and metabolic disorders after exposure to MPs [28]. Furthermore, the reproduction of *Magallana gigas* was negatively affected after the exposure [29]. In zebrafish, MPs also interfere with cell functions, leading to the production of reactive oxygen species (ROS), causing oxidative stress damage in organisms, and inducing changes in the expression of related genes [30]. As shown above, the current pollution problem of MPs is very serious.

In addition to their own toxic effects on organisms, MPs may also act as carriers of other pollutants in the environment, such as pharmaceuticals [31], heavy metals [32], and pesticides [33], thereby enhancing the enrichment and toxicity of these pollutants in aquatic organisms [34,35]. In marine pollution, 6.9% of samples contained cadmium (Cd), with concentrations exceeding 1 mg/g [36]. Cd is a non-degradable, carcinogenic, teratogenic, and mutagenic heavy metal. It is most commonly used in and released from electroplating industry, plastic industry, and nonferrous metal smelting. Even the metabolism of deep-sea mussel can be affected by Cd [37]. Cd can induce high levels of free radical production and thus accelerate cell apoptosis [38]. In addition, due to the accumulation effect and the enrichment effect of biological chains, even very low cadmium can have adverse effects on aquatic organisms [39]. The exposure and bioaccumulation of this substance have caused a variety of negative damages to amphibians, including developmental retardation, inadequate bone growth, and intestinal microbial community disorders [40,41,42].

New complex pollutants formed by MPs and heavy metals [43] may have multiple effects on organisms. First, MPs themselves can absorb heavy metals and be ingested by aquatic organisms, which may increase the concentration of heavy metals in them. Secondly, there may be superposition or synergy effects between MPs and heavy metals, and the interaction between the two may increase the toxicity of heavy metals. When MPs and heavy metals exist at the same time, it may be easier to pose a threat to the health of the organism. Some studies have found that MPs increase the Cd content in zebra fish gills, liver and intestines, and enhance the toxicity of Cd to zebrafish [44]. Barboza found that the addition of MPs not only increased the concentration of heavy metals in the gills and livers of *Dicentrarchus labrax* but also induced oxidative stress [45].

With globally and continually Amphibian populations decline, about 41% of species are at risk of extinction [46,47]. *Rana zhenhaiensis* (*R. zhenhaiensis*) belongs to the amphibian anura and is widely distributed in eastern China. Its living areas overlap with agricultural areas, making it vulnerable to man-made environmental pollution. Tadpoles have eggless shells, highly permeable skin, are found in large quantities, and are easy to be studied as environmental indicator species. At present, the research on the combined toxicity of MPs and heavy metals to amphibians is not sufficient. Whether the poisoning mechanism of the two pollutants on the researched tadpoles are synergistic or antagonistic needs further study. Therefore, this study took *R. zhenhaiensis* tadpoles as an environmental indicator species to explore the effects of combined exposure of typical heavy metal and plastics—Cd and polystyrene (PS) MPs on its growth, development and digestive system, and to assess its environmental and ecological risks.

## 2. Materials and Methods

### 2.1. Animals and Experimental Design

The fertilized eggs of *R. zhenhaiensis* were obtained from ponds near Tianhe Reservoir in Wenzhou, Zhejiang Province, China, and were incubated in dechlorinated tap water at room temperature. The background concentrations of Cd and MPs were <1 μg/L and 4.39 ± 1.13 items/L at the sample site, respectively.

Given that MPs in the environment cannot be completely avoided, we chose to use fluorescent MPs to distinguish them. Our preliminary experiments suggested that PS and PE caused more adverse effects on the development of tadpoles than PP. In addition, the majority of fluorescent MPs on the market are PS-MPs. Thus, fluorescent PS-MPs were chosen for the experiments. Fluorescent PS microspheres (grain diameter 1.5 μm) were purchased from Haian Zhichuan Battery Material Technology Co., Ltd., (Nantong, China). CdCl_2_·2.5H_2_O (AR, purity 99%) was purchased from Shanghai Jinshan Tingxin Chemical Reagent Factory (Shanghai, China). Based on the environmentally relevant concentration [48], and the 96 h LC_50_ (3413.5 μg/L) used for *R. zhenhaiensis* tadpoles (results of preliminary experiments), 50 μg/L Cd was used as the exposure concentration to ensure a safe concentration during chronic exposure. In addition, according to the studies by Limonta et al. [49] and da Costa Araújo et al. [50], 200 μg/L of PS was a moderate concentration, which is not much higher than that in the actual environment and can cause adverse effects in aquatic species. A stock solution of 2000 μg/L Cd was prepared by dissolved 0.2032 g of CdCl_2_·2.5H_2_O to 50 mL of deionized water. For the stock solution in 2000 μg/L PS, 0.2 g PS microspheres were dissolved into 100 mL of deionized water, and then the solution was dispersed for 30 min in ultrasonic bath at 45 MHz to prevent aggregation. Then, the working solution was prepared by dilution of the stock with dechlorinated tap water to obtain the target concentration 50 μg/L Cd and/or 200 μg/L PS.

When the tadpoles reached the Gosner [51] stage (Gs) 26 (begin to swim freely), semi-static exposure experiments (“control test”—“CT” with only dechlorinated tap water; “200 μg/L PS”—“PS”; “50 μg/L Cd”—“Cd”; “200 μg/L PS + 50 μg/L Cd”—“Cd_PS”) were performed in glass tanks with 4L dechlorinated tap water or working solution. Each treatment group was set up with 6 replicates, and each replicate contained 60 tadpoles. The tadpoles grew and developed in a ventilated laboratory with natural environment temperature, and a light and dark cycle of 12 h:12 h was guaranteed. To ensure water quality, the water in the tank was renewed by 2/3 every three days and the dead tadpoles were removed and counted during exposure. The tadpoles were fed with cooked lettuce, replacing the leaves daily and removing the tadpoles’ feces. The exposure was continued until the tadpoles had grown to Gs42 (metamorphic climax stage, forelimbs appear), which took about 60–80 days.

### 2.2. Cd and PS Analysis

Three tadpoles per group (*n* = 3/group) were randomly sampled from each treatment group at Gs42 and dissected after euthanizing with MS-222. The accumulation of PS in tadpoles was observed under an optical microscope (Nikon SMZ800N, Tokyo, Japan) with a UV Gooseneck lamp with a wavelength of 365 nm. Then, the liver and intestines were separated and weighed, and the Cd ion concentration was measured by AAS (Shimadzu, AA-7000F/G, Kyoto, Japan) using flame atomic absorption spectrometry (FAAS).

### 2.3. Measurement of Morphological Indicators

The growth indicators of Gs42 tadpoles (body weight, total length, head length, tibia length, tarsal length, hind limb length) were measured using an electronic balance (accuracy 0.0001 g) and a digital vernier caliper (accuracy 0.01 mm) after low-temperature anesthesia according to the guideline by Jiang and Li [52]. Twenty tadpoles were randomly selected from each treatment group for morphological indicator measurement. Subsequently, the tadpoles were subjected to bone double staining. Three tadpoles were selected from each treatment group and fixed with 4% paraformaldehyde. After 24 h, they were stored in 70% ethanol. The bone double staining method used two different dyes, Alizarin Red and Alizarin Blue, to make the hard tissue of the bones appear red and the soft tissue appear blue.

### 2.4. Histological Examination of Liver

Three tadpoles per group (*n* = 3/group) were randomly sampled from each treatment group at Gs42, euthanized with MS-222, dissected, and the liver was separated. The liver was fixed with 4% Bouin’s fixative for 12 h and then stored in 70% ethanol at 4 °C for histological analysis. Subsequently, the specimens were dehydrated by an ascending alcohol concentration, vitrified by dimethylbenzene, and embedded in paraffin. Thereafter, 7 μm thickness sections were cut and stained with HE. The stained slides were observed and photographed by light microscopes.

### 2.5. Transcriptomic Analysis and Quantitative Real Time-Polymerase Chain Reaction (RT-qPCR) of Liver

Nine tadpoles per group were randomly sampled at Gs42 and euthanized with MS-222. The livers were separated, and 3 livers were mixed into one sample (*n* = 3 mixed sample/group). The mixed liver samples were frozen in liquid nitrogen and then transferred to a −80 °C freezer to store. Total RNA was extracted from mixed samples using TRIzol Reagent (Invitrogen) and treated with DNase I (Takara Bio Inc., Shiga, Japan). RNA quality and quantity were assessed using a 2100 Bioana-lyzer (Agilent Technologies Inc., Santa Clara, CA, USA) and ND-2000 (NanoDrop Technologies, Waltham, MA, USA). Illumina HiSeq Sequencing li-braries were prepared from 1 μg of total RNA using the TruSeqTM RNA sample prep-aration Kit (Illumina, San Diego, CA, USA). Libraries were quantified with a TBS380 and sequenced on an Illumina NovaSeq 6000 sequencer (150 bp × 2, Shanghai BIOZERON Co., Ltd., Shanghai, China). Quality control was performed using Trimmomatic (version 0.36).

Data from all samples were subjected to RNA de novo assembly with Trinity. Assembled transcripts were BLASTX-searched against the NR, String, and Kyoto Encyclopedia of Genes and Genomes (KEGG) databases (E-value < 1.0 × 10^−5^). Gene Ontology (GO) annotations were obtained via BLAST2GO, and metabolic pathways were analyzed using KEGG.

Differential expression analysis between two conditions/groups using DESeq2 (1.20.0) was performed. DESeq2 provides a statistical program for determining differential expressions in digital gene expression data using a model based on negative binomial distribution. The obtained *p*-value was adjusted (padj) using the methods of Benjamin and Hochberg to control the error detection rate. Padj < 0.05&| log2 (foldchange) | > 1 is set as a threshold for significant differential expression.

### 2.6. Microbiota Analysis of Intestines

Eighteen tadpoles per group were randomly sampled at Gs42, euthanized with MS-222, dissected and separated the intestines. Three intestines were mixed into one sample (*n* = 6 mixed sample/group). After being washed with PBS, the mixed sample was used to extract the bacteria DNA with the E.Z.N.A.^®^ Stool DNA Kit (D4015-01, Omega Bio-tek, Norcross, USA). Then, the products were purified and quantified, following which, the normalized equimolar concentrations of amplicons were pooled and sequenced using an Illumina NovaSeq platform at the Novogene Bioinformatics Technology Co., Ltd. (Beijing, China).

### 2.7. Statistical Analysis

The statistical analysis of mortality, metamorphosis, and morphological data, including weight, total length, and Cd content, were performed using IBM SPSS 23 software (v23.0.0.0). Results were expressed as mean ± SD. Before conducting multiple comparisons, the Levene test was used to evaluate the homogeneity of variance, and the Kolmogorov–Smirnov normality test was used to check the normal distribution. One-way ANOVA and least significant range (LSD) were used for data analysis. Data with *p* < 0.05 were considered to have significant differences.

## 3. Results

### 3.1. Effects of Cadmium and Microplastic Exposure on the Growth of R. zhenhaiensis Tadpoles

The dechlorinated tap water exhibited the following characteristics: dissolved oxygen content—5.75 ± 0.37 mg/L; pH—7.2 ± 0.35; electrical conductivity—123.70 ± 7.41 μS/cm; and total dissolved solids—71.79 ± 3.69 mg/L. In addition, the Cd concentrations of CT group, PS group, Cd group, and Cd_PS group were 4.43 ± 2.87 μg/L, 5.70 ± 3.11 μg/L, 48.53 ± 3.11 μg/L, and 47.10 ± 4.80 μg/L, respectively.

On the 80th day, exposure to cadmium and microplastics alone or in combination significantly increased the mortality rate of tadpoles in the *R. zhenhaiensis* tadpoles (F_3, 20_ = 7.70, *p* < 0.05). The mortality rates of the CT group, PS group, Cd group, and Cd_PS group were 16.11 ± 6.02%, 28.61 ± 8.72%, 36.11 ± 4.91%, and 26.39 ± 8.72%, respectively. In addition, the metamorphosis rate of tadpoles significantly reduced after exposure Cd and PS alone or in combination (F_3, 20_ = 21.67, *p* < 0.05). The final metamorphosis rates of the CT group, PS group, Cd group, and Cd_PS group were 83.89 ± 6.02%, 71.39 ± 8.72%, 50.56 ± 4.91%, and 65.28 ± 8.72%, respectively.

**Figure 1:**
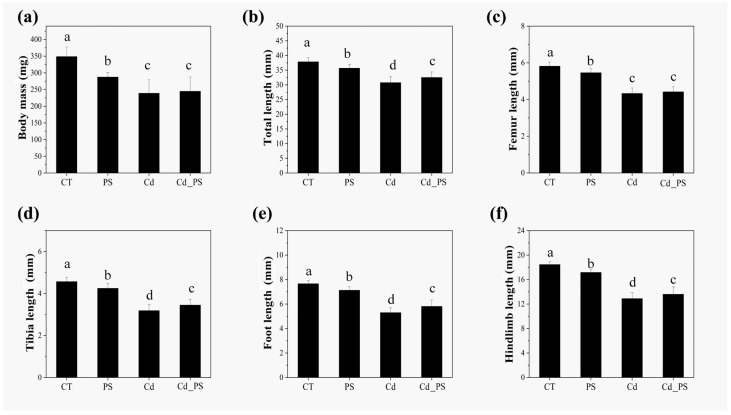
Effects of Cd and PS exposure on body mass (**a**), total length (**b**), femur length (**c**), tibia length (**d**), foot length (**e**), and hindlimb length (**f**) in tadpoles at Gs 42 of *R.zhenhaiensis.* The data are presented as mean ± SD. Differences between treatment groups are indicated by letter superscripts, with the same letter indicating a non-significant difference (*p* > 0.05) and different letters indicating a significant difference (*p* < 0.05) (a > b > c).

**Figure 2:**
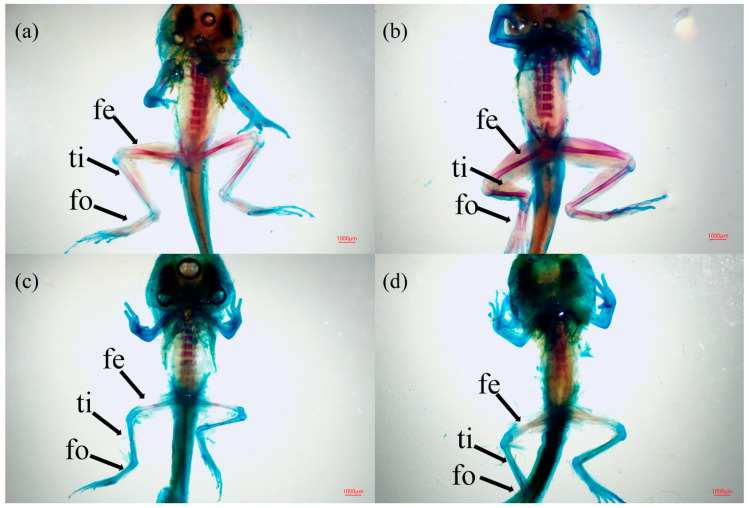
The skeleton of *R. zhenhaiensis* tadpoles at Gs 42. (**a**) CT group; (**b**) PS group; (**c**) Cd group; (**d**) Cd_PS group. Fe: femur; ti: tibia; fo: foot.

The effects of Cd and PS exposure on the morphological indices of tadpoles in the Gs 42 stage of the *R. zhenhaiensis* tadpoles are shown in Figure 1. The body mass of tadpoles at Gs 42 in the PS group, Cd group and Cd_PS group significantly decreased than that in the CT group (F_3, 76_ = 45.87, *p* < 0.05). In addition, compared with CT group, exposure to Cd and Cd_PS significantly decreased the body mass of tadpoles at Gs 42. The total length (F_3, 76_ = 71.55, *p* < 0.05), femur length (F_3, 76_ = 166.51, *p* < 0.05), tibia length (F_3, 76_ = 128.56, *p* < 0.05), foot length (F_3, 76_ = 166.26, *p* < 0.05), and hindlimb length (F_3, 76_ = 202.02, *p* < 0.05) of Gs 42 tadpoles in the PS group, Cd group, and Cd_PS group decreased more significantly than those of CT group. Compared to CT group, Cd (including Cd_PS) treatment caused the more severe inhibition in the body size of tadpoles. From the skeletal double staining (Figure 2), Cd and Cd_PS reduced the degree of skeletal ossification rate of femur and tibia in the tadpoles at Gs 42. The cartilage tissue (blue stained) did not undergo endochondral ossification to become hard bone (red stained). The untransformed cartilage bone tissue was mainly located at the ends of these long bones.

In summary, the individual or combined exposure of Cd and PS had significant negative effects on the development of mass, total length, and hind limbs in *R. zhenhaiensis* tadpoles at Gs 42.

### 3.2. Accumulation of Heavy Metals and Microplastics in R. zhenhaiensis Tadpoles

#### 3.2.1. The Accumulation of Cd in the Tadpoles

**Figure 3:**
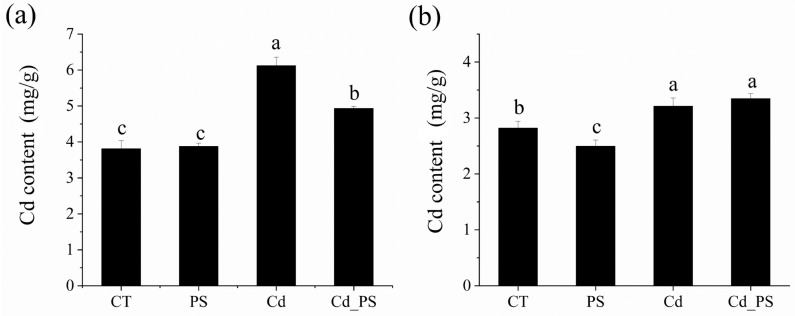
The Cd concentration in the (**a**) liver and (**b**) intestine. The data are presented as mean ± SD. Differences between treatment groups are indicated by letter superscripts, with same letter indicating a non-significant difference (*p* > 0.05) and different letters indicating a significant difference (*p* < 0.05) (a > b > c). *n* = 3.

The Cd content in the liver of the tadpoles in the Cd group was significantly higher than that of the other three groups (F_3,8_ = 119.03, *p* < 0.05) (Figure 3a). In addition, the Cd content in the liver of the Cd_PS group was significantly higher than that of the CT group and PS group. In intestines (Figure 3b), the Cd contents of tadpoles in Cd group and Cd_PS were significantly higher than those of the CT group and PS group (F_3,8_ = 30.95, *p* < 0.05).

#### 3.2.2. The Accumulation of PS in the Tadpoles

**Figure 4:**
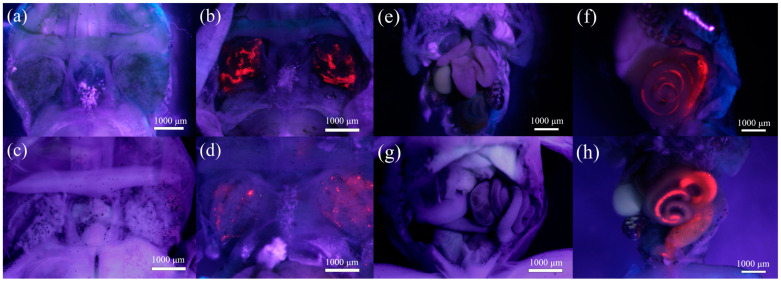
Accumulation of PS in the gills ((**a**): CT group; (**b**): PS group; (**c**): Cd group; (**d**): Cd_PS group) and the intestinal tract ((**e**): CT group; (**f**): PS group; (**g**): Cd group; (**h**): Cd_PS group) of *R. zhenhaiensis* tadpoles. PS was excited to fluoresce red.

After exposure to PS and Cd_PS, it can be seen from Figure 4 that there is obvious accumulation of PS particles (red fluorescence) in the gills and intestines of tadpoles. No significant fluorescence was observed in other visceral tissues.

### 3.3. Effects of Exposure to Cd and PS on Liver of R. zhenhaiensis Tadpoles

#### 3.3.1. Histological Observation of Liver

**Figure 5:**
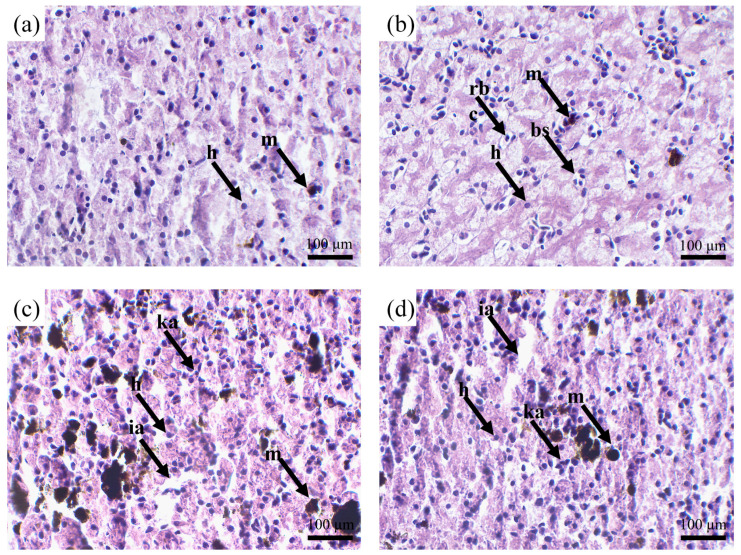
The effects of Cd and PS alone or exposed on the liver tissue structure of the Gs 42 stage *R. zhenhaiensis* tadpoles. (**a**) CT group; (**b**) PS group; (**c**) Cd group; (**d**) Cd_PS group. bs, blood sinus; h, hepatocyte; ia, intercellular space; m, melanomacrophage; rbc, red blood cell; ka, nuclear pyknosis.

The liver of tadpoles in different treatment groups was shown in Figure 5. The liver of tadpoles in CT group had normal cell structure with regular shape and boundary. The cytoplasm was evenly distributed, and a few melanomacrophages were seen. However, the liver of tadpoles in PS group showed dilated blood sinuses and in Cd group, as well as the Cd_PS group, it also showed increased melanomacrophages, nuclear pyknosis, and expanded cell intercellular space.

#### 3.3.2. Analysis of Differentially Expressed Transcriptome Genes and Oxidative Stress-Related Genes

RNA-seq was used to evaluate transcriptional levels in the liver of tadpoles at Gs 42. The results showed that compared with the CT group, the expression levels of 3420 genes in the PS group were significantly changed, of which 3122 genes were significantly up-regulated and 298 genes were significantly down-regulated. The expression levels of 9638 genes in group Cd were significantly changed, of which 6210 genes were significantly up-regulated and 3428 genes were significantly down-regulated. The expression levels of 11,554 genes in Cd_PS group were significantly changed, among which 7604 genes were significantly up-regulated and 3950 genes were significantly down-regulated.

**Figure 6:**
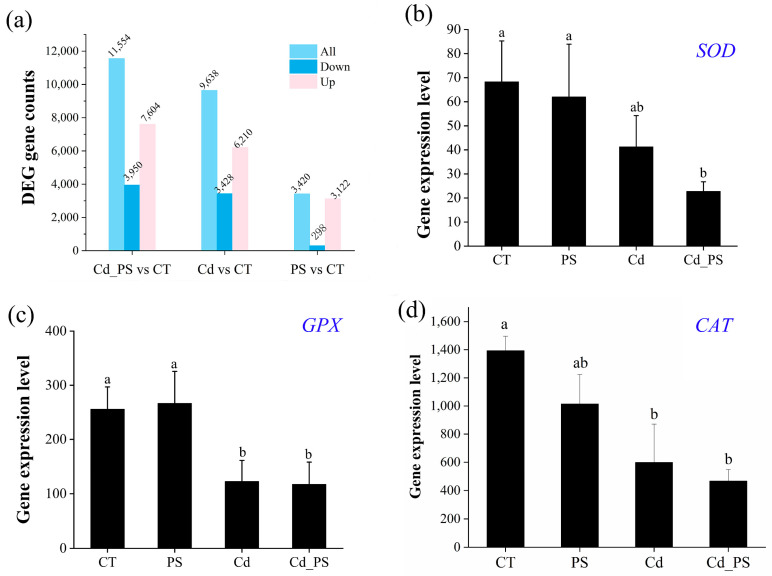
Statistics of the number of differentially expressed genes in differential comparison combinations. (**a**) Gene expression differences; (**b**) *SOD*; (**c**) *GPX*; (**d**) *CAT*. The gene expression levels are shown as FPKM. The data are presented as mean ± SD. Differences between treatment groups are indicated by letter superscripts, with the same letter or marker indicating a non-significant difference (*p* > 0.05) and different letters indicating a significant difference (*p* < 0.05) (a > b > c). *n* = 3.

The top 5 significant enrichment of KEGG pathways for differential gene were listed in Table S1. The functional prediction of genes in the liver of tadpoles in GO clustering, KOG clustering, and KEGG metabolic pathway classification were shown in Figures S1–S3, respectively. According to FPKM, the expression levels of oxidative stress-related genes were analyzed. As shown in Figure 6a, compared with the control group, the gene expression in PS group showed a downward trend, but no significant down-regulation was observed. However, exposure to Cd significantly down-regulated the expression levels of *GPX* (F_3,8_ = 3.01, *p* < 0.05) and *CAT* (F_3,8_ = 5.40, *p* < 0.05) in the liver of tadpoles. In addition, the expressions of *SOD* (F_3,8_ = 5.40, *p* < 0.05), *GPX* and *CAT* were significantly down-regulated in the liver of tadpoles in Cd_PS group.

### 3.4. Effects of Cd and PS Exposure on Intestinal Microorganisms of R. zhenhaiensis Tadpoles

#### 3.4.1. Effects of Cd and PS Exposure on the Alpha Diversity of Intestinal Microorganisms of *R. zhenhaiensis* Tadpoles

Venn diagrams (Figure S4) revealed that there were 84 shared OTUs among different exposure groups, and 839, 398, 160 and 792 unique OTUs were found in CT, Cd, PS, and Cd_PS groups, respectively. We use indices such as observed_otus, chao1, dominance, pielou_e, shannon, and simpson to assess intestinal microbial alpha diversity. Table 1 shows that PS and Cd exposure will reduce the total number of species (observed_otus), abundance (chao1), and evenness (pielou_e, shannon, and simpson) of the intestinal microbial communities to varying degrees. Compared with single exposure, combined exposure significantly increased gut microbial abundance (*p* < 0.05) and decreased species uniformity.

#### 3.4.2. Effects of Cd and PS Exposure on the Beta Diversity of Intestinal Microorganisms of *R. zhenhaiensis* Tadpoles

Unweighted unifrac distances are used to calculate the differences between differential ASVs and visualize them using principal coordinate analysis (PCoA). The principal coordinate combination with the largest contribution rate was selected to present the distribution of ASVs. As shown in Figure S5, the intestinal microbes of *R. zhenhaiensis* tadpoles in Cd group and Cd_PS group were significantly displaced compared with those in CT group. The composition and structure of community changed obviously. The intestinal microbial community of *R. zhenhaiensis* tadpoles in PS group was close to that of *R. zhenhaiensis* tadpoles in CT group, and the composition of microbial community was similar.

#### 3.4.3. Effects of Cd and PS Exposure on the Intestinal Microbial Community Composition of *R. zhenhaiensis* Tadpoles

**Figure 7:**
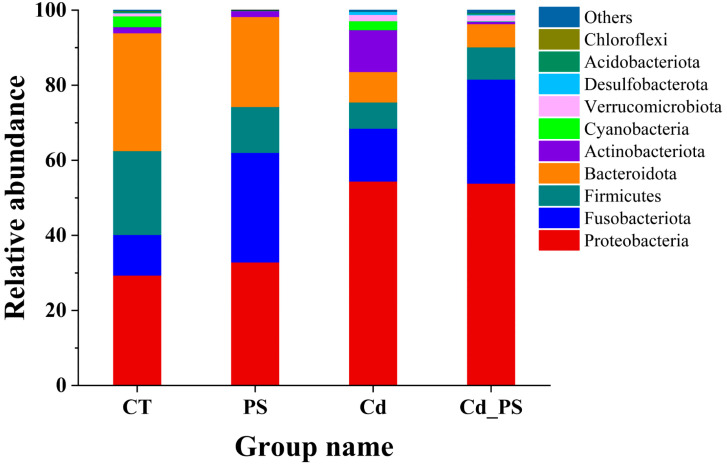
Top 10 gut microbiota species in phylum level.

The changes in intestinal microbiota abundance of *R. zhenhaiensis* tadpoles are shown in Figure 7. Based on species annotation, the top 10 species with the highest relative abundance at gate level were selected from each treatment group. It can be seen that Proteobacteria dominate the intestinal microbiota community of tadpoles. Compared with the CT group, the relative abundance of Proteobacteria in the Cd group and Cd_PS group increased. The relative abundance of Fusobacteriota in the PS group and Cd_PS group increased. The relative abundance of Firmicutes in the PS group, Cd group, and Cd_PS group decreased. The relative abundance of Bacteroidota in the PS group, Cd group, and Cd_PS group decreased. The relative abundance of Actinobacteriota in the Cd group increased.

## 4. Discussion

### 4.1. Accumulation of Cd and PS in Tadpoles of R. zhenhaiensis

The liver is a detoxification organ, which produces a large amount of metallothionein and Cd binding, so Cd concentration is the highest. For example, heavy metals would accumulate in organs such as the liver and intestine of organisms in *Cyprinus carpio* [53]. In the present study, the Cd ion content in the liver and intestine also had a high accumulation. Interestingly, after combined exposure, the accumulation of Cd in the liver was reduced; a possible reason for this is that some low-density PS adsorbed heavy metals to reduce the content of heavy metals in the liver, such as Cd adsorbed to PS in the intestine. The observation results showed that PS may aggravate the accumulation of Cd in the intestine, but it did not accumulate in the liver of *R. zhenhaiensis* tadpoles (Figure 3 and Figure 4). This was also confirmed in a study by Grigorakis et al. [54]. Microplastics may have the potential to adversely affect the intestine. This may be because microplastics that adsorb heavy metals are mainly digested in the intestine, resulting in the release of heavy metals [55]. Moreover, microplastics in the water enter tadpoles mainly through breathing/eating. Therefore, it accumulates the most in the intestine and gills. Some studies [18,56] have found that nanoscale microplastics can circulate to the whole body through the blood. In this study, the particle size of PS is slightly larger, so no systemic distribution is seen. In addition, microplastics may increase their concentration in the environment by adsorbing heavy metals, resulting in negative impacts on ecosystems and human health [32]. However, due to the wide range of microplastics with different particle sizes, types, and other characteristics, the adsorption of heavy metals by different types of microplastics may also vary, which in turn affects the accumulation of heavy metals in living organisms. There is still relatively little research on the combined toxic effects between microplastics and heavy metals, and the mechanism of this phenomenon is not yet clear, requiring further research.

### 4.2. Effects of Cd and PS on Tadpole Growth and Development

The morphologic indicators of metamorphosis rate and body size of amphibians are widely used biological indicators to assess the effects of chemical substances on amphibians. Cd and PS exposure significantly increased the mortality rate of tadpoles and decreased the metamorphosis rate and morphology of surviving tadpoles. According to the morphological indicators of the hind limb development of *R. zhenhaiensis* tadpoles in this study, the single or combined exposure of Cd and PS had significant negative effects on body weight, body length, and hind limb length of *R. zhenhaiensis* tadpoles in the Gs 42 stage. Exposure of Cd and PS alone or combined can affect amphibian development and reduce amphibian viability. The effect of single PS exposure on the morphology of tadpoles is smaller than that of single Cd exposure and combined exposure, and combined exposure has little difference from single Cd exposure in morphology. A large number of studies have shown that Cd has an effect on bones, which is also confirmed by bone staining.

Combined exposure did not increase or decrease the degree of bone ossification. Studies have shown that the accumulation of Cd may affect the thyroid gland, resulting in the inhibition of tadpole development [57]; bone is also considered to be the physiological target tissue of thyroid hormone, and bone ossification is regulated by thyroid hormone [58]. Therefore, we hypothesized that Cd and PS caused thyroid damage, resulting in reduced thyroid hormone secretion, which affected the growth and development of tadpoles.

In general, the growth and development indexes of the three toxic treatment groups were the highest in the PS treatment group and the lowest in the Cd treatment group, and the combined exposure group was between the two groups. By comparing the differences between groups, it can be concluded that Cd and PS are mainly toxic to the growth and development of *R. zhenhaiensis* tadpoles, and PS may inhibit the negative effects of Cd on the growth and development of *R. zhenhaiensis* tadpoles.

### 4.3. Effects of Cd and PS on Digestive System of Tadpoles

#### 4.3.1. Liver Histological Observation and Gene Expression

In the present study, compared to CT group, exposure to Cd group and Cd_PS group significantly caused structural damage to the liver of tadpoles of *R. zhenhaiensis*, including dilated blood sinuses, expanded cell intercellular space, and nuclear pyknosis. The single effect of Cd caused the greatest damage to liver tissue (Figure 5c), indicating the occurrence of hepatocyte apoptosis. Similar results also occurred in *Bufo gargarizans* larvae [40,41], where the introduction of Cd can reduce bone development, induce liver cell apoptosis, and cause liver tissue damage [41]. Compared to the Cd group, although PS can also cause dilated blood sinuses, the overall impact is relatively small (Figure 5b). Similar effects have also been observed in *Trachinotus blochii* [59] and *Physalaemus cuvieri* tadpoles [60]. The Cd_PS group had a relatively lesser poisoning effect compared to the Cd group, indicating that the combined exposure may have a weakened effect on cadmium exposure. This is different from the study of Zhang et al. [61], which demonstrated that MPs promote copper accumulation in the liver and pancreas of goldfish.

In vivo, the rise in reactive oxygen species (ROS) or the decrease in antioxidants can induce oxidative stress [62]. Oxidative stress can affect the assembly and operation of ribosomes, resulting in the disturbance of protein synthesis, which further affects protein synthesis and metabolism [63]. Catalase (CAT) and superoxide dismutase (SOD), as major antioxidant enzymes, can specifically remove intracellular superoxide anions, so as to avoid the damaging effect of ROS on the body [64]. Glutathione peroxidase (GPX) can reduce ROS levels by catalyzing glutathione to reduce hydrogen peroxide and other hydroperoxides to hydroxyl compounds [65]. The present study showed that compared with the CT group, the gene expression of *CAT* in the liver of tadpoles in the Cd exposure group was significantly down-regulated, and the expression of *SOD*, *GPX*, and *CAT* in the Cd_PS group were much more significantly down-regulated. It shows that the body produces oxidative stress, the detoxification ability decreases, and the homeostasis in the body is broken. It may be that PS and Cd have a combined effect, which inhibited the gene expression *SOD*, *GPX*, and *CAT,* exposing a more adverse effect on the body. Similar exacerbated result had been verified in the kidney of the mice that exposed to polystyrene fluorescent microspheres, polystyrene microspheres, and CdCl_2_ [66]. Early studies also showed that microplastics increased the concentration of heavy metals in the gills and livers of young fish, and also induced oxidative stress, which they believed may be due to the synergistic effect between microplastics and heavy metals in the liver [45]. Regardless of which experimental group, the pathological and gene expression changes in their liver tissues indicate that the function of the liver has been affected, which may be one of the reasons for the increased mortality rate and inhibited growth and development of *R. zhenhaiensis* tadpoles.

#### 4.3.2. Gut Microbes

In this study, the intestinal microflora of tadpoles in the combined exposure group was the least similar to that in the control group, indicating that the combined exposure of Cd and PS had a greater impact on the intestinal microflora. Both heavy metal and microplastic exposure affect the α diversity of gut microbes. Microplastic exposure reduced the total number of species, abundance, and uniformity of intestinal microflora. Cd exposure and combined exposure significantly decreased the uniformity of intestinal microflora compared with the control group, indicating that PS reduced the abundance and uniformity of intestinal microflora of tadpoles, while Cd and combined exposure reduced the uniformity of intestinal microflora of tadpoles. β diversity analysis (PCoA) helped to verify the above hypothesis. The displacement of intestinal flora in group Cd and group Cd.PS was negative, and that in group PS was positive. The displacement degree of Cd group was > Cd.PS group > PS group. The displacement degree of the first two groups was larger, indicating that the community structure had changed obviously. The intestinal microbial community of *R. zhenhaiensis* tadpoles in PS group was close to that of *R. zhenhaiensis* tadpoles in CT group, and the composition of microbial community was similar. The combined exposure of microplastics and heavy metals in blue killifish showed that microplastics alone caused a decrease in the diversity and abundance of the gut microbiota, and the combined exposure of microplastics and heavy metals further exacerbated this trend [67].

It is known that microbial communities are mainly controlled by Bacteroides, Proteobacteria, Firmicutes, and Fusobacteria, which are the four main categories of microorganisms in the intestinal ecosystem of amphibians [68,69]. In this study, the intestinal microbial community of *R. zhenhaiensis* tadpoles is also mainly composed of these four phyla. Cd exposure increased the relative abundance of Proteobacteria, and PS exposure increased the relative abundance of Fusobacteria. In addition, both changes could be found in the Cd_PS group. Similarly, Zhang et al. [70] suggested that exposure to 1250 mg/L of 10 μm sized PS-MPs increased the relative abundance of Fusobacteria but decreased Firmicutes in the *B. gargarizans* tadpoles. Interestingly, exposure to Cd increased the relative abundance of Bacteroides and decreased the Fusobacteria and Firmicutes in the *R. chensinensis* [69,71], as well as increased the relative abundance of Proteobacteria, Bacteroides, and Firmicutes, and decreased Fusobacteria in *B. gargarizans* tadpoles [42,72]. The different effects on gut microbiota in different tadpoles caused by Cd might be due to their different nutrient status and had inherently different microbiota. Recent studies suggest that an increase in the relative abundance of Proteobacteria may be a potential indicator of intestinal disorders [28,73]. The increased Proteobacteria in zebrafish’s intestine after exposure to only MPs might also generate more bacterial products, such as lipopolysaccharides (LPSs), which initiate inflammation, damage the intestinal mucosal barrier and increase intestinal permeability [28]. Fusobacteria have an “abridging” function within biofilms, contributing towards biofilm establishment and accumulation [69]. As the Cd has an inhibiting effect on it [69], the increase abundance of Fusobacteria in Cd_PS group mainly resulted from PS.

In addition, the relative abundance ratios of Bacteroidetes and Firmicutes are stable. The homeostasis of the intestinal microbiota is very important [56]. Changes in the relative abundance ratio of these two phyla can lead to instability of the microbial community, which can negatively affect the health of the host. Researchers have speculated that by keeping the relative abundance ratio of Bacteroides and Firmicutes stable, it could help maintain a stable gut microbiome, thereby promoting host health [74]. Therefore, the increase in the relative abundance of Proteobacteria induced by Cd and PS and the decrease in the ratio of Bacteroides to Firmicutes in this part of the study may indicate that exposure to Cd and PS may have adverse effects on *R. zhenhaiensis* tadpoles. And the combined exposure group caused more superimposed negative effects, which may be related to the properties of PS that absorb Cd and accumulate in the gut.

Moreover, other research has shown that only MPs fiber exposure could cause the declining abundance of Actinobacteria and might weaken the function of intestinal barrier and increase its sensitivity to immune stimulus [28]. In our research, although the Actinobacteria abundance was decreased after exposure to PS, it increased when exposed to Cd, and showed a declining trend in the Cd_PS group. The reason for the increase in Actinobacteria is not clear. The interaction between MPs and heavy metals is a complex subject, and MPs will likely influence heavy metal absorption, toxicity, accumulation, and transport [75]. According to Noreen Khalid et al. [35], as the guts/digestive systems of organisms have low pH, this could enhance the desorption of toxic metals, causing them to accumulate in organisms’ bodies. Therefore, the Cd_PS group showed a clear but complex additive effect in relation to the Cd and PS groups separately. However, at present, there are few studies on the combined toxicity of MPs and heavy metals to amphibians, as the mechanism is not clear, and further research is needed.

## 5. Conclusions

This study employed a series of experiments in the toxic effects of PS and Cd on the digestive system of *R. zhenhaiensis* tadpoles. The results show that PS and Cd can affect the normal physiological function of *R. zhenhaiensis* tadpoles. The compound effect of PS and Cd on the tadpoles’ morphological characteristics showed that the introduction of PS alleviated the effect of Cd. In addition, PS can significantly decrease the Cd accumulation in the liver but increase it in intestine. Although combined exposure reduces the accumulation of Cd in the liver, it does not reduce toxicity at the molecular level, and the effect is a combination of the two contaminants. Cd_PS had a cumulated influence on the gut microbiota of *R. zhenhaiensis* tadpoles. PS and Cd induced intestinal dysfunction in tadpoles, which may be related to the characteristics of microplastics which absorb heavy metals and accumulate in the intestine. This finding thereby provides a reference for further research on the toxicity mechanism of microplastics.

## Figures and Tables

**Table 1:** Alpha diversity index of gut microorganisms in *R. zhenhaiensis* tadpoles.

	CT	PS	Cd	Cd_PS
observed_otus	295.33 ± 205.25 ^ab^	133.83 ± 62.49 ^b^	233.50 ± 81.22 ^ab^	332.00 ± 80.85 ^a^
chao1	295.57 ± 205.45 ^ab^	134.07 ± 62.55 ^b^	234.27 ± 81.26 ^ab^	332.81 ± 81.01 ^a^
dominance	0.10 ± 0.04	0.15 ± 0.07	0.18 ± 0.08	0.20 ± 0.10
pielou_e	0.63 ± 0.06 ^a^	0.57 ± 0.07 ^ab^	0.52 ± 0.10 ^b^	0.49 ± 0.07 ^b^
shannon	4.90 ± 1.12	3.92 ± 0.41	4.03 ± 0.93	4.09 ± 0.71
simpson	0.90 ± 0.04	0.85 ± 0.07	0.82 ± 0.08	0.80 ± 0.10

Note: Differences between treatment groups are indicated by letter superscripts, same letter or no letter indicates a non-significant difference (*p* > 0.05) and different letters indicate a significant difference (*p* < 0.05) (a > b > c).

## Data Availability

The data of this study are available upon reasonable request to the corresponding authors.

## Supplementary Materials

The following supporting information can be downloaded at: https://www.mdpi.com/article/10.3390/toxics12120854/s1. Table S1: Differential gene analysis; Figure S1: Function prediction of liver genes of *R. zhenhaiensis* tadpoles using GO clustering; Figure S2: Functional prediction of liver genes of *R. zhenhaiensis* tadpoles using KOG clustering; Figure S3: Functional prediction of liver genes of *R. zhenhaiensis* tadpoles using GO clustering KEGG metabolic pathway classification statistical map; Figure S4: Venn Graph of *R. zhenhaiensis* gut microbiota; Figure S5: PCoA diagram based on Unweighted Unifrac distance.
