# Supplementary material for: Toxicity of Polystyrene Microplastics with Cadmium on the Digestive System of Rana zhenhaiensis Tadpoles
Source: Toxics. 2024 Nov 26;12(12):854. doi: 10.3390/toxics12120854 (PMC11679246; doi:10.3390/toxics12120854)
Supplement: Supplementary file 1 [file toxics-12-00854-s001.zip › toxics-3307079-supplementary.pdf]

Table S1. Differential gene analysis

Analysis groups	*Pathway differences*	*Differential gene*
Cd vs CT	U	Ribosome、PI3K-Akt signaling pathway、Phagosome、MAPK signaling pathway and Proteoglycans in cancer are the most significantly upregulated KEGG pathway, with 94, 41, 37, 35, and 33 DEGs enriched in it, respectively.
	D	Ribosome、Protein processing in endoplasmic reticulum、Complement and coagulation cascades、Chemical carcinogenesis and Ferroptosis are the most significantly downregulated KEGG pathway, with 48, 42, 31, 21, and 20 DEGs enriched in it, respectively.
PS vs CT	U	Metabolic pathways、Biosynthesis of secondary metabolites、Microbial metabolism in diverse environments、Valine, leucine and isoleucine degradation and two-component system are the most significantly upregulated KEGG pathway, with 106、38、25 and 10 DEGs enriched in it, respectively.
	D	not significant
Cd_PS vs CT	U	Ribosome、Thermogenesis、Oxidative phosphorylation、Parkinson disease and Proteoglycans in cancer are the most significantly upregulated KEGG pathway, with 96、51、38、38 and 33 DEGs enriched in it, respectively.
	D	Complement and coagulation cascades、Chemical carcinogenesi、Staphylococcus aureus infection、Proteasome and Metabolism of xenobiotics by cytochrome P450 are the most significantly downregulated KEGG pathway, with 46、27、24、22 and 21 DEGs enriched in it, respectively.
Cd_PS vs Cd	U	not significant
	D	Complement and coagulation cascades, Staphylococcus aureus infection are the most significantly downregulated KEGG pathway, with 14 and 11 DEGs enriched in it, respectively.
Cd_PS vs PS	U	Ribosome, Thermogenesis, Parkinson disease, Proteoglycans in cancer and Oxidative phosphorylation are the most significantly upregulated KEGG pathway, with 75、34、26、25 and 23 DEGs enriched in it, respectively.

	D	Metabolic pathways、Biosynthesis of secondary metabolites、Microbial metabolism in diverse environments、 Biosynthesis of amino acids and Drug metabolism - other enzymes are the most significantly downregulated KEGG pathway, with 143、56、38、 19 and 15 DEGs enriched in it, respectively.

*Upregulation: U; Downregulation: D

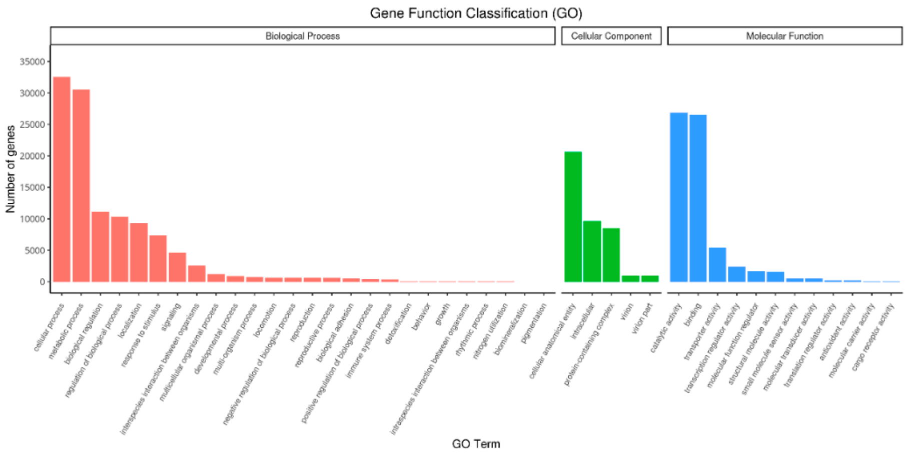

Figure S1. Function prediction of liver genes of *R. zhenhaiensis* tadpoles using GO clustering.

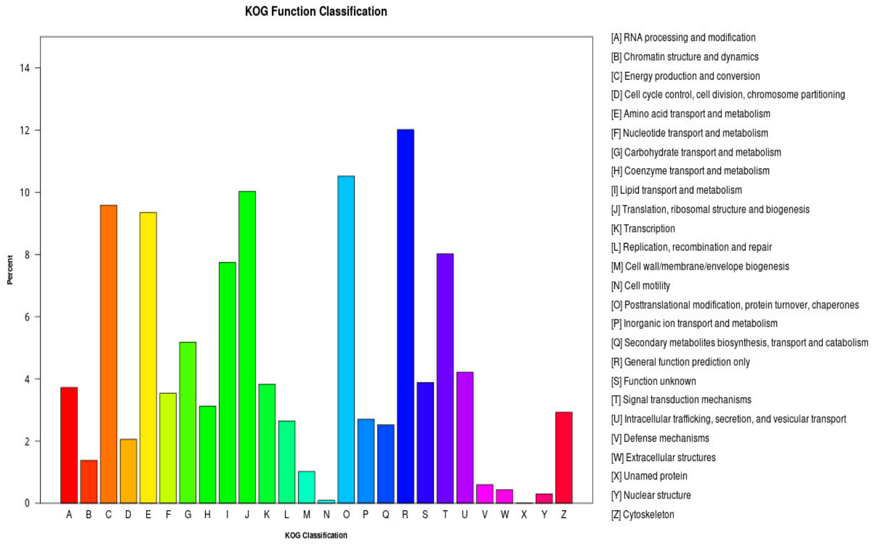

Figure S2. Functional prediction of liver genes of *R. zhenhaiensis* tadpoles using KOG clustering.

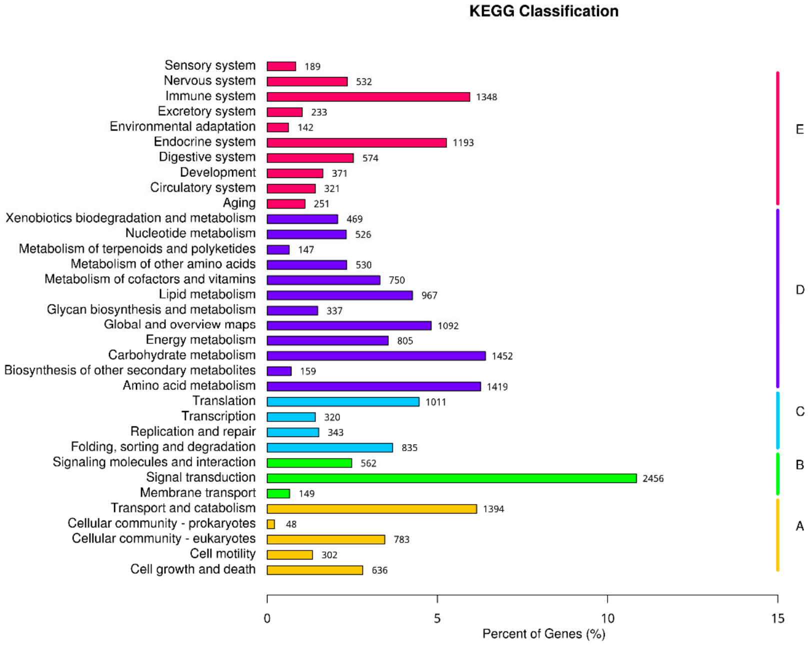

Figure S3. Functional prediction of liver genes of *R. zhenhaiensis* tadpoles using GO clustering

KEGG metabolic pathway classification statistical map

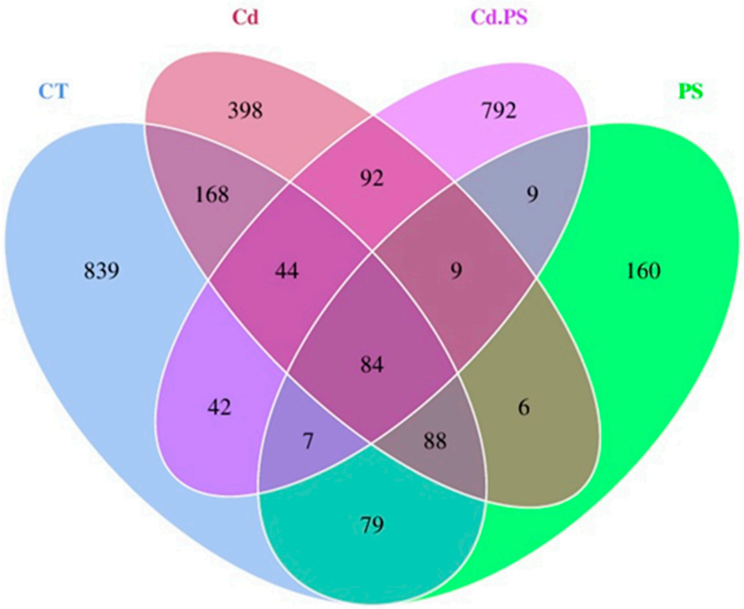

Figure S4. Venn Graph of *R. zhenhaiensis* gut microbiota

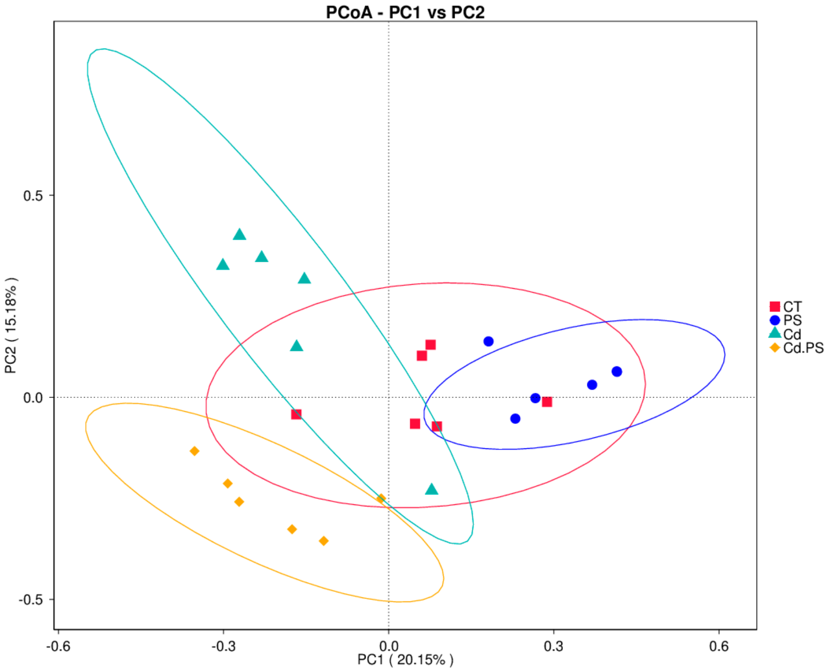

Figure S5 PCoA diagram based on Unweighted Unifrac distance
